# Impact of Long Non-coding RNAs Associated With Microenvironment on Survival for Bladder Cancer Patients

**DOI:** 10.3389/fgene.2020.567200

**Published:** 2020-11-12

**Authors:** Gaoteng Lin, Baoyin Guo, Yulei Wei, Tianjie Lan, Simeng Wen, Gang Li

**Affiliations:** ^1^Department of Urology, Tianjin Institute of Urology, The Second Hospital of Tianjin Medical University, Tianjin, China; ^2^Department of Urology, Tianjin Baodi Hospital, Baodi Clinical College of Tianjin Medical University, Tianjin, China; ^3^Department of Gynecology and Obstetrics, Tianjin First Central Hospital, Tianjin, China

**Keywords:** bladder cancer, long non-coding RNA, immune score, microenvironment, nomogram, prognosis

## Abstract

**Background:**

Cumulative evidence from several tumor studies, including bladder cancer, emphasizes the importance of the tumor microenvironment (TME) in tumorigenesis, development, and metastasis, which can be regulated by long non-coding RNAs (lncRNAs). This study aims to identify bladder cancer (BC) microenvironment–associated lncRNAs for their prognostic value predicting the survival of BC patients.

**Methods:**

The data of BC patients regarding lncRNA expression and corresponding clinical characteristics were obtained from The Cancer Genome Atlas (TCGA). The Cox regression analysis and the least absolute shrinkage and selection operator (LASSO) regression analysis were performed to screen lncRNAs following the calculation of the immune score for each sample. For the screened lncRNAs, a risk score model was constructed to predict the survival, and 3- and 5-year overall survival (OS) rates were assessed using a nomogram. The calibration curve and concordance index (C-index) validated the performance of the nomogram. Finally, to explore the potential function related to the screened lncRNAs, gene ontology (GO) and Kyoto Encyclopedia of Genes and Genomes (KEGG) enrichment analysis were performed.

**Results:**

The multivariate Cox regression analysis screened five TME-associated lncRNAs regarded as independent factors influencing the tumor progression. The corresponding risk score model was established as follows: (−0.15816 AC064805.1) + (0.10015 AC084033.3) + (−0.17977 AC092112.1) + (−0.05673AC103691.1) + (0.17789 AL391704.1) + (−0.16258 LINC00892). The C-index for the nomogram was 0.63 (95% CI: 0.625–0.635). Also, the calibration curve verified the predictive effectiveness by showing a good concordance between the nomogram prediction and the actual observation. GO and KEGG analysis demonstrated that six TME-associated lncRNAs were most likely linked to tumor metastasis and progression.

**Conclusion:**

The present study determined six lncRNAs as independent immuno-biomarkers in the TME, constructed a nomogram to predict their prognostic value, and investigated the potential biological processes to understand their regulatory roles in the progression of BC.

## Introduction

Due to poor prognosis and high recurrence, bladder cancer (BC) is considered a significant threat to male health (Cumberbatch et al., 2018). Concerning the mortality rates, BC ranks 13th, affecting up to 165,000 cases worldwide (Cumberbatch et al., 2018). For histological typing, staging, and stopping recurrence, transurethral resection of the bladder (TURB) is the most preferred method in the management of non-muscle-invasive bladder cancer (NMIBC) (Babjuk et al., 2017). Also, during disease management combined with chemotherapy or immunotherapy, close follow-up after patient discharge can effectively reduce the risk of tumor recurrence and progression to muscle-invasive bladder cancer (MIBC) (Babjuk et al., 2017). In some patients, an early radical cystectomy could be beneficial in the case of non-metastatic MIBC, but the corresponding comprehensive treatment scheme must be applied to improve the prognosis in a case-specific manner (Alfred et al., 2017). However, regardless of vigorous intervention measures, the final treatment outcome of MIBC is mostly unfavorable. Recently, antitumor fibroblast growth factor receptor (FGFR) targeting agents have shown promising results in clinical trials (Nogova et al., 2017). Apart from the traditional treatment strategy, novel U.S. FDA-approved immune checkpoint inhibitors are now being suggested as a first-line and metastatic treatment for BC (Rouanne et al., 2016). Similarly, identifying the key biomarkers linked to the tumor regulatory network could facilitate early diagnosis and timely targeted treatment to reduce the risk of recurrence, progression, and mortality in BC.

Long non-coding RNAs (lncRNAs) are transcribed from the non-coding regions that are ∼75% of the genome exposed to frequent genomic mutations (Djebali et al., 2012). Overcoming the experimental challenges, high-throughput sequencing technologies and functional tracking permit a profound understanding of alternation in the structure and function of lncRNAs caused by persistent genomic mutations. These changes disrupt the intracellular equilibrium of the regulatory network, giving rise to various cellular activities, including tumor cell transformation (Huarte, 2015). Prostate cancer–associated 3 (PCA3) (Bussemakers et al., 1999) and prostate cancer gene expression marker 1 (PCGEM1) (Srikantan et al., 2000) were the first discovered cancer-associated lncRNAs. PCA3 facilitates prostate cancer progression and tumor cell proliferation via regulation of the miR-218-5p/HMGB1 pathway (Zhang et al., 2019) and is also considered to be a molecular diagnostic biomarker in clinical practice (Hessels et al., 2003). PCGEM1, by activating transcription, regulates the expression of androgen receptor 3, which leads to castrate-resistant prostate cancer (Yang et al., 2013; Zhang et al., 2016). Likewise, the intracellular regulatory roles of several lncRNAs among other tumors have also been examined. For instance, MALAT1 in lung cancer (Gutschner et al., 2013), H19 in colorectal cancer (Ren et al., 2018), HULC in liver cancer (Xin et al., 2018), UCA1 in bladder cancer (Xue et al., 2017), and PVT1 in renal cancer (Ren et al., 2019) are the known diagnostic biomarkers in respective cancers and are under investigation for the targeted treatment.

In various tumors, the infiltrating immune cells are heterogeneous in nature. Therefore, the clinical outcomes and prognosis are closely linked to the level and types of immune cells in the local tumor site (Fridman et al., 2012). It is reported that lncRNAs can regulate the immune response by interacting with genomes, chromatin, RNA, and proteins and have also been linked to the differentiation and activation of immune cells, such as T cells and myeloid cells (Gomez et al., 2013; Hu et al., 2013; Wang et al., 2014). Therefore, to find specific immuno-biomarkers, it is important to examine the role of immune cells in tumor development and infiltration from the perspective of lncRNA-mediated regulation.

Recently, several studies screened for lncRNAs to develop relevant models for the prognosis of corresponding tumors (Cai et al., 2019; Miao et al., 2019). Furthermore, the prognostic value can be assessed based on the components involved in the progression of tumors, such as immune genes and immune cells. However, there are rare studies on tumor microenvironment (TME)-associated lncRNAs that may indirectly regulate tumorigenesis and progression of BC. Here, we screened six TME-associated lncRNAs, evaluated their prognostic value, and investigated potential function. These findings pave the way to look for novel immuno-biomarkers for diagnosis and immunotherapy to decrease recurrence and drug resistance in BC.

## Materials and Methods

### Data Acquisition Relevant to BC

The Cancer Genome Atlas (TCGA)^1^ database was used to retrieve the lncRNA expression profiles related to BC patients (*n* = 433) and clinical characteristics (*n* = 412) that included age, gender, stage, grade, and TNM staging. Also, the immune scores of the corresponding samples (*n* = 408) were obtained from the Estimation of STromal and Immune cells in Malignant Tumor tissues using Expression data (ESTIMATE).^2^ Data lacking missing or unknown values were excluded.

### Screening of TME-Associated lncRNAs for Prognosis

Using the lncRNA expression profiles and the corresponding immune score, the BC samples were distinguished into either the high or low immune score group based on the median immune score. The sample sizes of the groups were comparable; 206 cases belonged to the high immune score group and 208 cases formed the low immune score group. The “limma” R software package (version 3.6.2) was utilized to screen the differentially expressed lncRNAs (DElncRNAs) with the inclusion criteria of log2-fold change >1 and false discovery rate (FDR) <0.05. The univariate Cox regression analysis, the least absolute shrinkage and selection operator (LASSO) regression analysis, and the multivariate Cox regression analysis were performed to identify the independent TME-associated lncRNAs. These models were used to analyze the influence of multiple independent variables on a dependent variable and were a major method for screening independent factors. Kaplan–Meier analysis and log-rank tests were employed to determine the survival-related lncRNAs. Then, the risk score model for lncRNAs was built to estimate survival risk for each patient as follows:

$$\mathrm{Risk}\,\mathrm{score}=\sum_{i=1}^{N}\left({\text{E}}_{\text{i}}*\beta_{\text{i}}\right)$$

Here, N, E_*i*_, and β_*i*_ denote the number of selected lncRNAs, the expression level of the ith lncRNA, and the ith lncRNA coefficient, respectively. Receiver operating characteristic (ROC) analysis was used to estimate the sensitivity and specificity of the prognosis-related lncRNAs. *P* < 0.05 was considered as statistical significance.

### Constructing a Predictive Survival Model

Combined with the clinical characteristics, a nomogram consisting of age, gender, stage, immune score, and risk score was established to predict the 3- and 5-year overall survival (OS) probability for BC patients. The nomogram was validated using the calibration curve and the concordance index (C-index).

### Assessing the Immune Cell–Specific Expression and the Potential Function of the Six lncRNAs

To further examine the expression of the selected lncRNAs in specific immune cells, the promoters and enhancers targeting the selected lncRNAs were obtained using the Human Gene database.^3^ Then, functional enrichment analysis was performed to assess the biological process and functional pathway related to these six lncRNAs along with analysis of coexpressed mRNAs. Pearson correlation coefficient analysis was utilized to find the correlation between the lncRNAs and the protein-coding genes. Using the threshold Pearson correlation coefficient >0.10 and *p* < 0.01, a total of 2,764 protein-coding genes were selected for gene ontology (GO) and Kyoto Encyclopedia of Genes and Genomes (KEGG) analysis in the Metascape project^4^ (Zhou et al., 2019). The GO interaction networks and KEGG pathways were visualized using the Cytoscape software (Shannon et al., 2003).

## Results

### DElncRNAs

**FIGURE 1:**
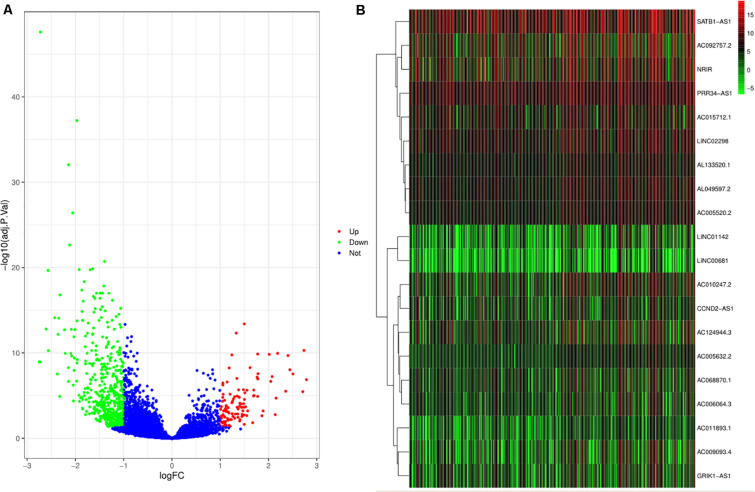
Differentially expressed lncRNAs (DElncRNAs) and lncRNAs with immune score. **(A)** The volcano plot depicts the expression level of 10933 lncRNAs. The green, red, and blue dots imply downregulated and upregulated lncRNAs and no differential expression, respectively. **(B)** The heat plot shows lncRNAs with immune score. The top half of the heat plot refers to the top 10 expressing lncRNAs with a high immune score. Likewise, the bottom half of the heat plot refers to the top 10 expressing lncRNAs with a low immune score.

After excluding the missing and unknown data, the remaining relevant data related to clinical characteristics used in this study are presented in Supplementary Table 1. Out of 10,933 lncRNAs, a total of 627 DElncRNAs are shown in Figure 1A. Combined with the immune score, the top 10 DElncRNAs with a high immune score and the other top 10 DElncRNAs with a low immune score are presented in Figure 1B.

### LncRNAs as the Independent Prognostic Factors Based on the Risk Score Model

**FIGURE 2:**
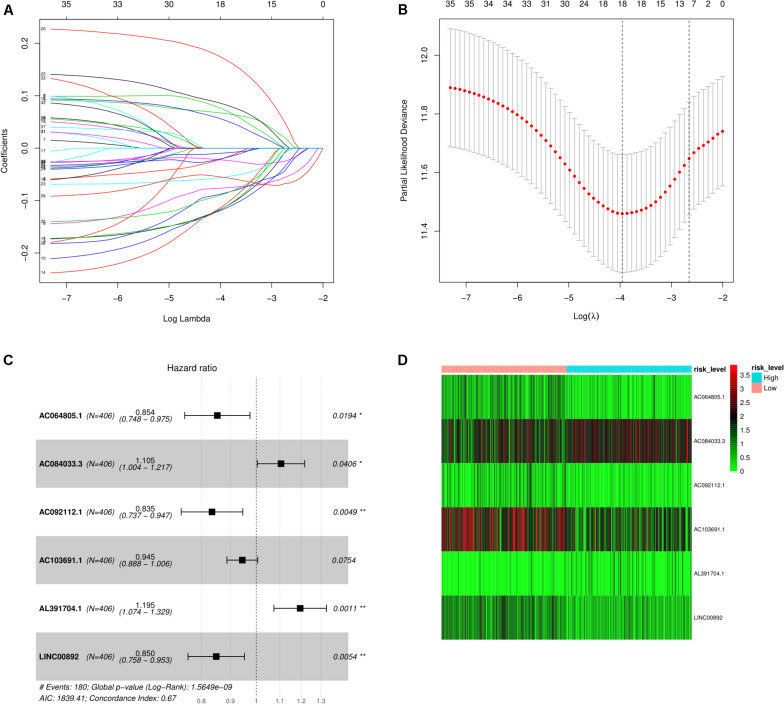
Screening of lncRNAs as independent prognostic factors. **(A)** The LASSO regression model was utilized to validate the parameter selection adjustment. **(B)** The distribution of LASSO coefficient profiles for lncRNAs as prognostic factors. **(C)** Based on the multivariate Cox regression analysis, the forest plot shows lncRNAs with independent prognostic value. **(D)** The heat plot depicts the low- and high-risk expression profiles of the six lncRNAs in BC samples.

The results of the univariate Cox regression analysis of prognosis-associated independent lncRNAs (*p* < 0.05) are exhibited in Supplementary Table 2. Then, to avoid overfitting in the final model, LASSO regression analysis was performed on the results obtained from the univariate Cox regression analysis as shown in Figures 2A,B. Finally, six lncRNAs were identified for the subsequent multivariate Cox regression analysis. These are displayed using a forest plot in Figure 2C. The results demonstrate that AC064805.1 (HR: 0.854; 95% CI: 0.748–0.975; *P* = 0.0194), AC092112.1 (HR: 0.835; 95% CI: 0.737–0.947; *P* = 0.0049), and LINC00892 (HR: 0.850; 95% CI: 0.758–0.953; *P* = 0.0054) with prolonged OS probability were regarded as independent positive prognostic factors. On the contrary, AC084033.3 (HR: 1.105; 95% CI: 1.004–1.217; *P* = 0.0406) and AL391704.1 (HR: 1.195; 95% CI: 1.074–1.329; *P* = 0.0011) with the higher mortality were identified as independent negative prognostic factors. Based on the risk score and multivariate Cox regression analysis coefficients, the predicted OS for the six lncRNAs is as follows: (−0.15816 AC064805.1) + (0.10015 AC084033.3) + (−0.17977 AC092112.1) + (−0.05673AC103691.1) + (0.17789 AL391704.1) + (−0.16258 LINC00892). Using the median risk score, the BC samples were separated into either a high- or a low-risk score group as exhibited in Figure 2D.

### Correlation of the Five lncRNAs With Overall Survival

**FIGURE 3:**
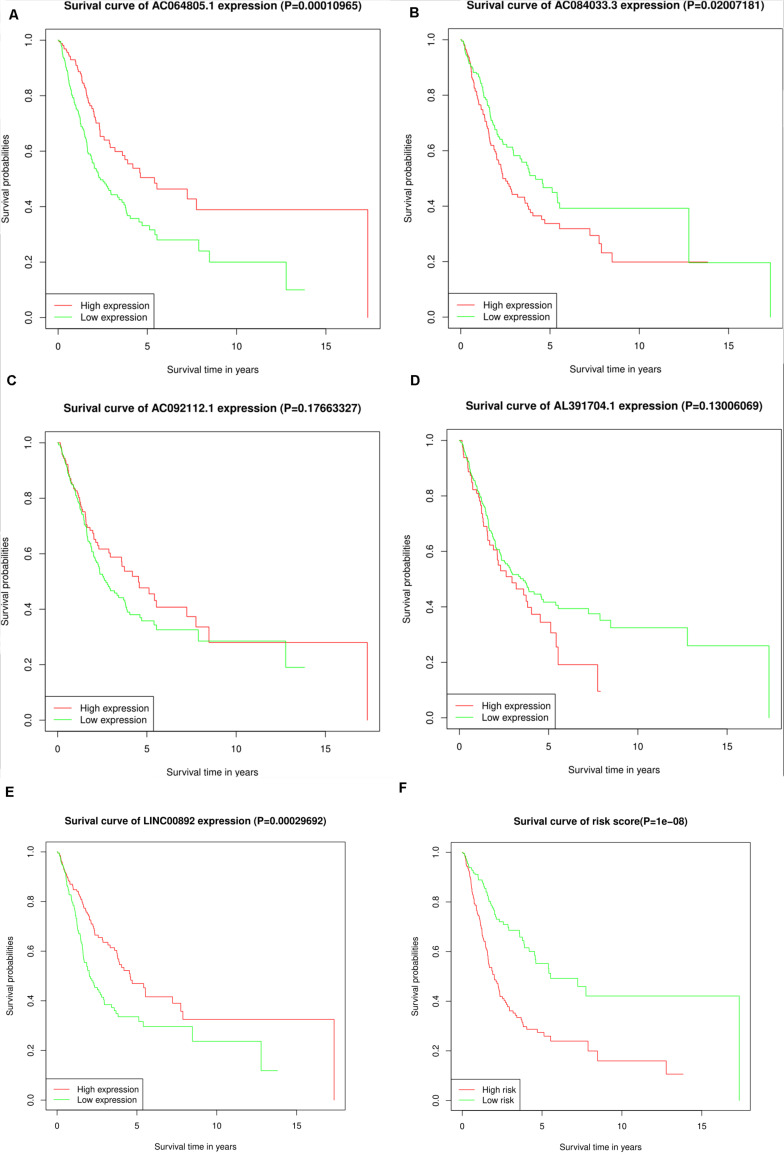
KM survival analysis for the five lncRNAs and the risk score. **(A–F)** Kaplan–Meier curves of OS for AC064805.1, AC084033.3, AC092112.1, AL391704.1, LINC00892, and risk score, respectively.

Depending on the median expression level, the individual expression level of the five lncRNAs ranged from high to low expression levels. Kaplan–Meier analysis was used to find the correlation between the five lncRNAs and the overall survival rates (Figures 3A–E). We found that the high expression of AC064805.1 and LINC00892 were related to prolonged OS; however, high expression of AC084033.3 was associated with an unfavorable OS rate. The survival analysis of the risk scores is displayed jointly in Figure 3F. A high-risk score denotes an increased risk of mortality.

### Prediction of OS Rates Using a Nomogram

**FIGURE 4:**
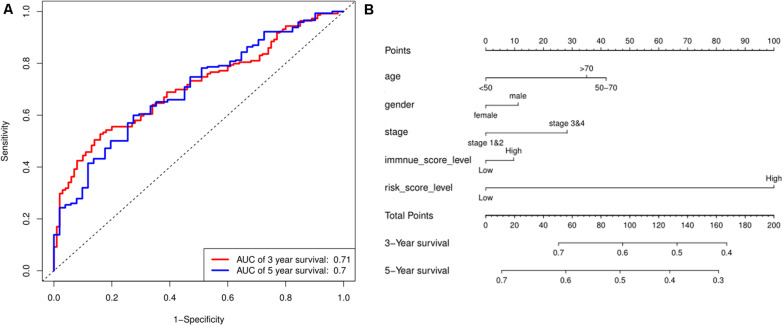
Prediction for OS. **(A)** ROC curve shows the sensitivity and specificity of the risk score model for predicting 3- and 5-year OS probability. **(B)** Each variable was scored according to the patient’s status, and the total score was obtained by adding each variable score. The vertical line represents the corresponding 3- and 5-year OS probability prediction.

**FIGURE 5:**
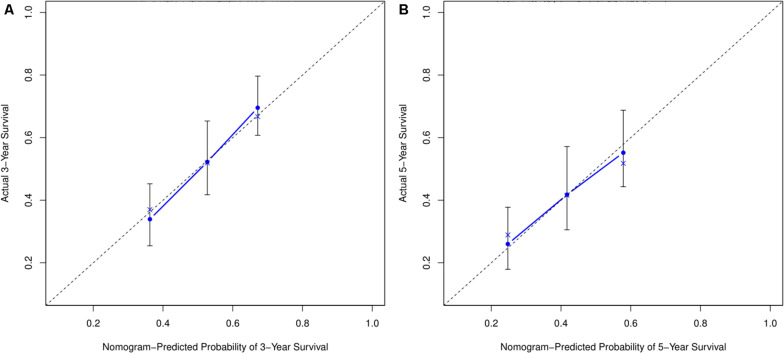
Calibration curve for nomogram prediction. **(A,B)** 3- and 5-year OS prediction performed by the nomogram. A small gap between the blue solid and black dotted lines demonstrates good performance of the nomogram.

Based on the risk score model, the predicted 3- and 5-year OS rates of the six lncRNAs were subjected to ROC analysis as demonstrated in Figure 4A. The area under the ROC curve for the predicted 3- and 5-year OS was 0.70 and 0.71, respectively, emphasizing the effectiveness of the model in predicting the OS rates. Next, the risk score; the immune score; and the clinical characteristics such as age, gender, and stage were incorporated to construct a nomogram to predict the 3- and 5-year OS for BC patients as displayed in Figure 4B. The nomogram was validated using the concordance index (C-index) and the calibration curve. The C-index was 0.63 (95% CI: 0.625–0.635). Also, the calibration curve revealed good concordance between the prediction and the actual observation, indicating the predictive effectiveness of the nomogram as illustrated in Figures 5A,B.

### Expression of Selected lncRNAs in Specific Immune Cells and Investigation of the Potential Function of the Six lncRNAs

**FIGURE 6:**
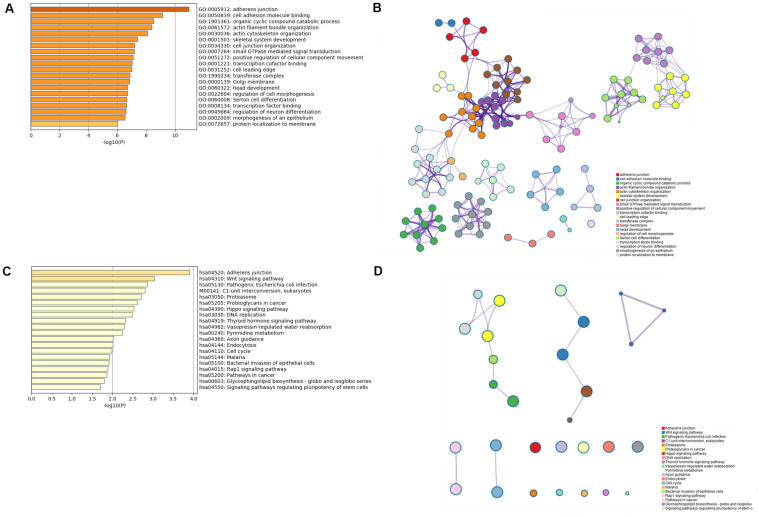
GO and KEGG analysis for the six lncRNAs coexpressed with mRNAs. **(A,B)** The bar plot and interaction network plot for the top 20 enrichment of GO analysis. **(C,D)** The bar plot and interaction network plot for the top 20 enrichment of KEGG analysis.

The results from the database about data analysis related to specific immune cells demonstrated that some promoters and enhancers could specifically target the genes to selectively transcribe the lncRNAs enhancing their expression as presented in Supplementary Table 3. Then, a total of 2,764 protein-coding genes coexpressing with the six lncRNAs were subjected to the GO and KEGG analysis using the Metascape platform. From GO functional annotation, we retrieved the top 20 significantly enriched GO terms, which are shown in Figure 6A. The six lncRNAs were found to be associated with several biological processes, including GO:0005912∼adherens junction, GO:0050839∼cell adhesion molecule binding, GO:1901361∼organic cyclic compound catabolic process, etc. The interaction network of the top 20 enriched GO terms were visualized as shown in Figure 6B. Also, KEGG pathway analysis demonstrated that the genes coexpressing with the lncRNAs were majorly enriched in hsa04520: Adherens junction, hsa04310: Wnt signaling pathway, M00141: C1-unit interconversion, eukaryotes, etc. The top 20 KEGG pathways are shown in Figure 6C. GO and KEGG analysis demonstrated that six TME associated lncRNAs were potentially related to tumor metastasis and progression. Correspondingly, the interaction network for the top 20 KEGG pathway is displayed in Figure 6D.

## Discussion

Over the recent decade, a vast majority of tumor studies focused on the influence of biological processes and relevant pathways related to oncogenes, tumor suppressor genes, and their products to understand tumor development, progression, and metastasis. However, considerable tumors, such as BC are heterogenic in nature with a high mortality rate. This has compelled researchers to focus on the impact of the TME, especially the associated lncRNAs affecting the OS of tumor patients (Fridman et al., 2012; Huarte, 2015; Bhan et al., 2017). The status and expression levels of diverse infiltrating immune cells affect the biological processes of tumors, leading to distinct clinical outcomes (Fridman et al., 2012). The lncRNAs are known to affect the state of infiltrating immune cells by regulating the related biological processes or pathways. The lncRNAs CCAT1 and NIFK-AS1 alter the polarization of M2 macrophages by regulating the expression of miRNA to modulate the infiltration of macrophage subtypes in TME and expedite tumor progression and invasion (Zhou et al., 2018; Liu et al., 2019). These studies suggest that lncRNAs can be utilized as biomarkers of macrophage polarization and potential drug targets for immunotherapy in the corresponding tumors. The lncRNAs are also known to modulate the biological processes related to T cells, such as activation, development, and differentiation (Heward and Lindsay, 2014). Upregulated lnc-Tim3 and lnc-sox5 destroy the balance of the TME by decreasing the infiltration of antitumor CD8 + T cells aiding tumor progression (Wu et al., 2017; Ji et al., 2018). lnc-EGFR via regulating AP-1/NF-AT1/Foxp3 signaling pathway and lncRNA SNHG1 by regulating miR-448/IDO affect the differentiation and growth of immunosuppressive regulatory T cells (Tregs) and enable immune escape for tumor (Jiang et al., 2017; Pei et al., 2018). Similarly, the dendritic cell differentiation is modulated by lnc-DC via activation of transcription factor STAT3, weakening the antitumor response (Wang et al., 2014). Concisely, all these findings indicate that lncRNAs play an essential role in regulating the activities of immune cells in TME, further emphasizing the relationship between tumor heterogeneity and immune cell infiltration in a variety of tumors. Notably, tumor exosome–derived lncRNAs have been shown to enable specific communications between tumor cells and immune cells. For example, tumor cell–derived lncRNA TUC339 activates macrophages to modulate the macrophage cytokine production, phagocytosis, M1/M2 polarization, and cell proliferation (Li X. et al., 2018). It further strengthens the notion that lncRNAs may influence the infiltration of immune cells to regulate their functions, which are closely related to tumor growth, progression, and metastasis. Therefore, lncRNAs can indeed serve as novel immuno-biomarkers in the corresponding tumors.

Because several lncRNAs are involved in tumor growth, progression, and metastasis, a risk score model was established to comprehensively consider their role in prognosis. The utility of LASSO regression analysis identified six TME-associated lncRNAs, and the multivariate Cox regression analysis recognized five lncRNAs as potential immuno-biomarkers and therapeutic targets. The ROC score for the six lncRNAs for 3- and 5-year OS was predicted to be 0.70 and 0.71, respectively. This model can also be utilized to evaluate the prognostic values of the multiple lncRNAs. Finally, a nomogram was developed to attain the individual prognosis information based on patient-specific conditions. These results could help clinicians in the early prognosis of the disease and to timely intervene reducing the mortality rate in BC patients.

Almost 75% of the genome can produce lncRNAs, which are now being explored as tumor hallmarks (Djebali et al., 2012). They exist in TME and may also influence other tumor hallmarks, including the immune system. Cancer cell–derived lncRNA H19 targets endothelial cells to promote angiogenesis by modulating the production and release of VEGF, contributing to tumor growth (Conigliaro et al., 2015). Exosomal lncRNA PTENP1 inhibits tumor progression by regulating PTEN expression via binding to microRNA-17 (Zheng et al., 2018). lncRNA FAL1 binds to miR-1236 to promote tumor cell proliferation and metastasis (Li B. et al., 2018). In addition, lncRNAs are also associated with antitumor drug resistance. For instance, sunitinib resistance is the result of altered expression of AXL and c-MET caused by lncARSR binding to miR-34/miR-449 (Qu et al., 2016). In this study, mRNA coexpression analysis was carried out to investigate the potential function of the six lncRNAs. The enrichment analysis was conducted using GO and KEGG analysis. The results suggest that these lncRNAs could be involved in critical cellular processes, such as tumor metastasis, catabolism, cell cycle, DNA replication, and so on.

Nonetheless, there are limitations to our study. First, the TCGA database is limited to constructing a risk score model and nomogram. Therefore, additional databases should be utilized to validate the six lncRNA signatures and the nomogram performance. Second, only limited clinical characteristics were included in the nomogram to uphold the accuracy of the prediction. Third, because the several lncRNAs have not been fully validated yet, the selected lncRNAs lack laboratory evidence. Using the database, we could only infer about the expression of the six lncRNAs in specific immune cells. Fourth, the associated biological processes and pathways need to be validated experimentally to investigate the molecular mechanism revealing the characteristics of the TME-associated lncRNAs.

## Conclusion

In a nutshell, our study screened six BC microenvironment–associated lncRNAs, identified independent risk factors influencing OS, and constructed models to predict the prognostic value. Also, functional enrichment analysis revealed that the six lncRNAs were most likely related to BC metastasis and progression. However, further experiments are required to investigate the associated pathways.

## Data Availability Statement

The data used and analyzed during the current study are available from TCGA (https://portal.gdc.cancer.gov/).

## Author Contributions

GLi, SW, and GLin: conception and design. GLin, BG, YW, and TL: data collection, data analysis and interpretation. GLin and GLi: manuscript writing. All authors: final approval of manuscript.

## Conflict of Interest

The authors declare that the research was conducted in the absence of any commercial or financial relationships that could be construed as a potential conflict of interest.

## Footnotes

1 https://portal.gdc.cancer.gov/

2 https://bioinformatics.mdanderson.org/estimate/disease.html

3 https://www.genecards.org/

4 http://metascape.org
