# Supplementary material for: Impact of Long Non-coding RNAs Associated With Microenvironment on Survival for Bladder Cancer Patients
Source: Front Genet. 2020 Nov 12;11:567200. doi: 10.3389/fgene.2020.567200 (PMC7689372; doi:10.3389/fgene.2020.567200)
Supplement: Supplementary file 1 [file Table_1.docx]

Supplemental Table 1: clinical characteristics

Characteristics Total percentage

sample size 376

Age

<50 22 5.9%

50-70 186 49.5%

>70 168 44.6%

Gender

female 98 26.1%

male 278 73.9%

Stage

Ⅰ 2 0.5%

Ⅱ 104 27.7%

III 140 37.2%

IV 130 34.6%

T

T1 3 0.8%

T2 118 31.4%

T3 196 52.1%

T4 59 15.7%

N

N0 223 59.3%

N1 46 12.2%

N2 74 19.7%

N3 7 1.9%

NX 26 6.9%

M

M0 180 47.9%

M1 8 2.1%

MX 188 50%
