# Supplementary material for: Impact of Long Non-coding RNAs Associated With Microenvironment on Survival for Bladder Cancer Patients
Source: Front Genet. 2020 Nov 12;11:567200. doi: 10.3389/fgene.2020.567200 (PMC7689372; doi:10.3389/fgene.2020.567200)
Supplement: Supplementary file 2 [file Table_2.docx]

Supplemental Table 2：The results of the univariate Cox regression analysis (p<0.05)

lncRNA HR P

ADAMTS9-AS1 1.051492378 0.043567164

AL354950. 0.95701699 0.03659457

AC083949.1 0.929170783 0.046368103

ST20-AS1 0.928596327 0.031010975

LINC01281 0.899244753 0.025301095

AC103691.1 0.905179798 0.000478543

TRBV11-2 0.881926713 0.022571138

AC022613.1 1.070305878 0.049457152

AC106801.1 0.853408654 0.021657272

LINC02446 0.940578984 0.038529555

AC073655.2 0.922458795 0.025866001

AC022126.1 0.872188898 0.027089576

AC034238.1 0.861639047 0.038703582

AC104261.1 0.813340419 0.013724752

SAMSN1-AS1 0.82645182 0.014835932

PCAT29 0.873454447 0.034498271

HCP5 0.918078741 0.005992298

AC026369.3 0.890651431 0.018515652

LINC00158 0.836611298 0.008754397

AL160400.1 0.914968378 0.049168627

LINC01914 0.915519354 0.0394797

LINC02245 0.82455216 0.030156337

LINC01736 0.963829928 0.041080877

AC096558.1 0.823725366 0.018193561

LINC00892 0.799582665 3.12E-05

LINC01871 0.938705071 0.037748592

AC092112.1 0.863532546 0.011930053

AC104078.1 0.812285041 0.019851807

AL391704.1 1.125466259 0.01942949

AC064805.1 0.792684066 8.76E-05

CHRM3-AS2 0.875285064 0.001413832

AL023653.1 0.887985053 0.045042238

LINC01215 0.939680928 0.046633512

AC007389.5 0.87970762 0.030531997

AC084033.3 1.122676324 0.008409149

AP005131.2 0.871015146 0.034457365

LINC00705 1.119634543 0.016204657
