# Supplementary material for: Impact of Long Non-coding RNAs Associated With Microenvironment on Survival for Bladder Cancer Patients
Source: Front Genet. 2020 Nov 12;11:567200. doi: 10.3389/fgene.2020.567200 (PMC7689372; doi:10.3389/fgene.2020.567200)
Supplement: Supplementary file 3 [file Table_3.docx]

Supplemental Table 3: the expression of selected lncRNAs in specific immune cells

Target gene promoter enhancer immune cells

AC064805.1 [GH17J074600](http://genecards.weizmann.ac.il/geneloc-bin/display_map.pl?chr_nr=17&range_type=gh_id&gh_id=GH17J074600#GH17J074600) [GH17J074600](http://genecards.weizmann.ac.il/geneloc-bin/display_map.pl?chr_nr=17&range_type=gh_id&gh_id=GH17J074600#GH17J074600) monocytes

[GH17J074598](http://genecards.weizmann.ac.il/geneloc-bin/display_map.pl?chr_nr=17&range_type=gh_id&gh_id=GH17J074598#GH17J074598)

[GH17J074569](http://genecards.weizmann.ac.il/geneloc-bin/display_map.pl?chr_nr=17&range_type=gh_id&gh_id=GH17J074569#GH17J074569) [GH17J074569](http://genecards.weizmann.ac.il/geneloc-bin/display_map.pl?chr_nr=17&range_type=gh_id&gh_id=GH17J074569#GH17J074569)

[GH17J074612](http://genecards.weizmann.ac.il/geneloc-bin/display_map.pl?chr_nr=17&range_type=gh_id&gh_id=GH17J074612#GH17J074612)

[GH17J074606](http://genecards.weizmann.ac.il/geneloc-bin/display_map.pl?chr_nr=17&range_type=gh_id&gh_id=GH17J074606#GH17J074606)

AC092112.1 [GH12J014216](http://genecards.weizmann.ac.il/geneloc-bin/display_map.pl?chr_nr=12&range_type=gh_id&gh_id=GH12J014216#GH12J014216) [GH12J014216](http://genecards.weizmann.ac.il/geneloc-bin/display_map.pl?chr_nr=12&range_type=gh_id&gh_id=GH12J014216#GH12J014216) natural killer cell, T-cell, B cell

[GH12J014221](http://genecards.weizmann.ac.il/geneloc-bin/display_map.pl?chr_nr=12&range_type=gh_id&gh_id=GH12J014221#GH12J014221) CD14-positive monocyte, T cell, natural killer cell

LINC00892 [GH0XJ136640](http://genecards.weizmann.ac.il/geneloc-bin/display_map.pl?chr_nr=0X&range_type=gh_id&gh_id=GH0XJ136640#GH0XJ136640) [GH0XJ136640](http://genecards.weizmann.ac.il/geneloc-bin/display_map.pl?chr_nr=0X&range_type=gh_id&gh_id=GH0XJ136640#GH0XJ136640) T-cell

[GH0XJ136671](http://genecards.weizmann.ac.il/geneloc-bin/display_map.pl?chr_nr=0X&range_type=gh_id&gh_id=GH0XJ136671#GH0XJ136671) T-cell, natural killer cell

[GH0XJ136663](http://genecards.weizmann.ac.il/geneloc-bin/display_map.pl?chr_nr=0X&range_type=gh_id&gh_id=GH0XJ136663#GH0XJ136663) monocytes

AC103691.1 [GH15J064954](http://genecards.weizmann.ac.il/geneloc-bin/display_map.pl?chr_nr=15&range_type=gh_id&gh_id=GH15J064954#GH15J064954) B cells

AC084033.3 unknown unknown unknown

AL391704.1 unknown unknown unknown
